# Skin Disease Profile in Geriatric Inpatients at a Tertiary Referral Hospital

**DOI:** 10.21315/mjms2023.30.6.6

**Published:** 2023-12-19

**Authors:** Lili Legiawati, Shannaz Nadia Yusharyahya, Rinadewi Astriningrum, Amanda Andria Pulungan, Ratih Wulan Kusumahapsari

**Affiliations:** Department of Dermatology and Venereology, Dr. Cipto Mangunkusumo Hospital, Faculty of Medicine Universitas Indonesia, Jakarta, Indonesia

**Keywords:** dermatitis, geriatric, infection, skin disease

## Abstract

**Background:**

Elderly people are susceptible to various skin diseases. To monitor disease trends in the geriatric population, epidemiologic data on skin diseases are essential. However, studies on skin diseases in geriatric patients in Indonesia, particularly those who are hospitalised, are limited. Therefore, this retrospective study aims to determine the skin disease profile in geriatric patients at a tertiary referral hospital in Indonesia.

**Methods:**

The subjects were all geriatric inpatients who were consulted at the Department of Dermatology and Venereology, Dr. Cipto Mangunkusumo National General Hospital between 2017 and 2019. The patients were analysed according to sex, age and comorbidities. They were divided into 15 groups according to the diseases. Chi-square test or Fisher’s exact test was used to compare categorical variables.

**Results:**

The most common skin diseases were infections (35.8%), dermatitis (21.8%), ulcers (12.8%), age-related skin changes (8.4%) and vascular diseases (5.3%). Dermatitis was significantly more common in males (*P* < 0.05), whereas infections were significantly more common in females (*P* < 0.05).

**Conclusion:**

Consistent with previous studies, our inpatient data showed that infections were significantly more common in females, whereas dermatitis was significantly more common in males. The data collected may contribute to epidemiologic data on skin diseases in the geriatric population.

## Introduction

The elderly is defined as people aged 60 years old or older (1). In recent years, the geriatric population has increased significantly throughout the world, including Indonesia (2, 3). The global population aged 60 years old and older is expected to almost double from 12% to 22% between 2015 and 2050 (2). Indonesia has entered the period of population aging due to the increase in life expectancy and geriatric population. In 2010, the number of elderly people in Indonesia was approximately 18 million (7.56%). This number is expected to increase to 25.9 million (9.7%) in 2019 and 48.2 million (15.77%) in 2035 (3).

Aging leads to a decline in organ function, including the skin. In aging skin, barrier function, wound healing, cell renewal capacity, thermoregulation, immune responsiveness, sensory perception, DNA repair capacity, and sebum and sweat production are decreased. Several structural changes occur in aging skin, including wrinkling and roughness (4). Apart from morphological changes, the elderly population is more susceptible to various skin diseases (5).

Diagnosing and treating certain skin diseases can be challenging for clinicians because geriatric patients often have comorbidities and multiple medications (6). In addition, the clinical manifestations of skin diseases in the elderly may not be as typical as those in younger people (5). Skin diseases in the geriatric population in each country vary. A retrospective study in India found that erythemato-squamous diseases were the most common skin diseases, followed by skin infections, parasitic infestations and pruritus (7). In Indonesia, a study in an outpatient dermatology clinic found that dermatitis, skin tumour, cosmetic disorders, infectious disease and xerosis cutis were the five most common skin diseases (8).

Many skin diseases have been shown to affect the quality of life of the elderly population. Thus, epidemiological data on skin diseases are essential to monitor disease trends in the geriatric population (6). However, studies on skin diseases in Indonesian geriatric patients, particularly those who are hospitalised, are limited. Therefore, this study aimed to determine the skin disease profile of geriatric patients hospitalised at the Dr. Cipto Mangunkusumo National General Hospital, a tertiary referral hospital.

## Methods

This retrospective study was conducted at the Dr. Cipto Mangunkusumo National General Hospital, Indonesia. The subjects were all geriatric inpatients who were consulted at the Department of Dermatology and Venereology, Dr. Cipto Mangunkusumo National General Hospital between 2017 and 2019. Patient data based on daily shift reports during 2017–2019 were collected. All inpatients aged 60 years old or older who were consulted at the Department of Dermatology and Venereology were included and those who were not consulted were excluded. The study was approved by the Health Research Ethics Committee, Faculty of Medicine, Universitas Indonesia and the Dr. Cipto Mangunkusumo National General Hospital.

A total of 235 patients were analysed according to sex, age and comorbidities. The patients were divided into 15 groups based on diseases, namely, infections (including secondary infections), dermatitis, ulcers, pruritus, vascular diseases, age-related skin changes, erythrosquamous dermatosis, pigmentation disorders, benign neoplasms, autoimmune vesiculobullous disease, drug eruptions, metastatic skin tumours and malignant skin neoplasm, pyoderma, amyloidosis and sweat duct disorders.

### Statistical Analysis

Categorical variables were compared using chi-square test or Fisher’s exact test. Statistical significance was considered at *P* < 0.05. All statistical analyses were performed using SPSS version 25.0.

## Results

A total of 235 patients were enrolled in this study. Among them, 119 (50.6%) were female and 116 (49.4%) were male. According to the age groups, 149 (63.4%), 65 (27.7%) and 21 (8.9%) patients were in the 60 years old–69 years old, 70 years old–79 years old and ≥ 80 years old of age groups, respectively. Table 1 shows that respiratory diseases were the most common comorbidities in geriatric patients with skin diseases (48.9%), followed by renal and urinary tract diseases (31.1%), heart diseases (29.4%) and type 2 diabetes mellitus (T2DM) (24.7%).

The most common skin disorders were infections (35.8%), dermatitis (21.8%), ulcers (12.8%), age-related skin changes (8.4%) and vascular disease (5.3%) (Table 2). The most common infections were cutaneous candidiasis (11.7%), secondary infections (8.7%) and tinea (faciei, corporis, cruris, pedis and manus) (6.1%). The most common types of dermatitis were irritant contact dermatitis (13.7%), seborrheic dermatitis (3.8%) and intertriginous dermatitis (1.5%). The most common ulcers were pressure ulcers (8%), traumatic ulcers (2%), and bacterial ulcers (1.5%). Our study found that the most common age-related skin disorder was xerosis cutis (7.9%). The most common vascular diseases were senile purpura (2.3%) and extravasation injury (1.2%). Erythrosquamous dermatoses consisted of psoriasis (-guttata, -inversa, -vulgaris, -scalp, -sebo), erythroderma, pityriasis sicca, pityriasis rosea and generalised pustular psoriasis. However, only psoriasis cases were found in this group (two subjects).

Infection, dermatitis, ulcer and age-related skin changes were the four most common diseases within two different age groups. However, in the 60 years old–69 years old and 70+ years old of age groups, the fifth most common disease was vascular disease and autoimmune vesiculobullous disease, respectively (Table 2). Dermatitis was significantly more common in males (*P* < 0.05), whereas infection was significantly more common in females (*P* < 0.05) (Table 3).

## Discussion

In our study, infection was the most common disease (35.8%), with cutaneous candidiasis being the most common infection (11.7%). This finding is similar to that in previous studies (7, 9, 10). A study from India found a higher incidence of infections in the elderly (29.9%), with fungal infection being the most common infection (18%) (7). T2DM was the fourth most common comorbidity. Based on our previous study, we found that T2DM can cause dry skin (75.61% of cases), which occurs mainly on the feet, leading to a higher risk of infection (11). Diabetic patients also tend to be more prone to infections, including primary pathogen and fungal infections (12). Delamaire et al. (13) showed that elevated blood glucose levels may affect granulocyte function and cause tissue invasion, which may promote superficial fungal growth on the skin of diabetic patients. In addition, some studies suggest that several factors such as weakened immune system, decreased blood flow, and dryness lead to delayed healing process in geriatric patients, which may lead to infection (14). Moreover, the result of the previous and our study reflects the high humidity in both countries.

The present study also showed that infection was significantly more common in females than in males. Teklebirhan et al. (15) and Balakumar et al. (16) showed that females were more affected by fungal infections than males. However, in these studies, dermatophytosis was the type of fungal infection observed. In our study, 16 of 31 female patients with cutaneous candidiasis had T2DM. A similar result was reported in a previous study, wherein the presence of *Candida* infection was common in females (17). The study found that skin surface pH in the axillary and inguinal regions was significantly higher in diabetic patients compared with healthy subjects. In addition, 12% of subjects had *Candida* in the intertriginous area. Elevated skin surface pH in the intertriginous regions of diabetic patients may provide an environment in which *Candida* can develop (17).

Dermatitis is common in the geriatric population. The frequency of irritant contact dermatitis and seborrheic dermatitis was 13.7% and 3.8%, respectively. This finding is similar to that of Bilgili et al. (18), wherein contact dermatitis and seborrheic dermatitis were the most commonly seen dermatitis. Another retrospective study conducted at a neurosurgical unit found that dermatitis and infection accounted for more than half of all dermatology consultations. The most common dermatitis found in the patients was contact dermatitis (11.3%). In addition, most patients were in the 60 years old–79 years old of age group (19). In our study, dermatitis was significantly more common in males than in females. This finding is consistent with that of a Turkish study, which found that dermatitis was significantly more frequent in males than in females (18). Another study also showed that the odds ratio for occupational allergic contact dermatitis is five times greater in males than in females and found that dermatitis was common among construction workers and janitors (20). However, in our study, information regarding the patient’s occupation was not obtained.

During the aging process, several degenerative and metabolic changes occur in the skin, making the elderly more susceptible to skin diseases. Contributing factors include systemic diseases, health and hygiene, socioeconomic status, climate, gender, diet, culture and personal habits such as smoking (5). Elderly skin is thinner, more translucent and undergoes elastosis, making it more fragile. As the skin ages, the normal number of epidermal stem cells is maintained, but the ability to migrate and respond to proliferative signals is reduced. These factors, along with primary immune system defects, lead to barrier defects and make dermatitis more common in the elderly population. In addition, due to the impaired immune system, the elderly often have chronic inflammation, referred to as inflammaging. Inflammaging is thought to be the result of the remodeling of the innate and adaptive immune systems, leading to the chronic production of inflammatory cytokines that reduce the skin’s ability to defend against pathogens. The presence of pruritus from the dermatitis and infection itself, combined with decreased sensory perception in geriatric patients, especially those with diabetes, may provoke the onset of more severe infections (21).

Xerosis is one of the most common complaints in the geriatric population. In a previous study, xerosis was significantly more common in males than in females and in the 65 years old–74 years old of age group (18). Decreased sebum secretion in the elderly may lead to xerosis and skin dryness may increase without the application of emollients (22). However, in the current study, there were no statistical differences between xerosis and gender/age. The differences in results may be due to different climatic conditions, comorbidities and medications.

The incidence of psoriasis increases among the geriatric population (23, 24). Psoriasis may cause psychosocial disability and impaired quality of life in the elderly due to its chronic relapsing nature (25). In our previous study, the age range of the elderly with psoriasis was between 60 years old and 79 years old (26). Another study found that psoriasis was more common in the 65 years old–74 years old of age group (18). In this study, psoriasis was found in both age groups. However, the result may not be representative of the population with psoriasis due to the small number of subjects. Another factor that should be considered is that all patients with psoriasis in our previous study received narrowband UVB phototherapy (26).

The main limitation of this study is that our data were based on inpatients who were consulted in the Department of Dermatology and Venereology. Therefore, geriatric patients with skin diseases who were not consulted were not included in this study. In addition, because only pre-existing data were analysed, further information on the onset and course of the disease could not be obtained. Thus, the association between diseases and age and sex may have little clinical relevance. However, there are few studies that have evaluated skin diseases in hospitalised geriatric patients in Indonesia. This study could help raise awareness of skin diseases in the geriatric population and help physicians in diagnosing skin diseases in the elderly.

The elderly are an important part of the population. Consistent with previous studies, our inpatient data showed that infection was significantly more frequent in females, whereas dermatitis was significantly more frequent in males. The data collected may contribute to epidemiological data on skin diseases in the geriatric population. Future studies with larger sample size and involving associated factors are needed to investigate skin diseases in geriatric populations.

## Figures and Tables

**Table 1:** Distribution of age group and comorbidities in study subjects according to gender

Characteristics	Gender		Total number of cases (%)

	Male (%)	Female (%)	
Age groups (years old)			
60–69	80 (34)	69 (29.4)	149 (63.4)
70–79	31 (13.2)	34 (14.5)	65 (27.7)
≥ 80	8 (3.4)	13 (5.5)	21 (8.9)
Total			
Comorbidities	119 (50.6)	116 (49)	235 (100)
Respiratory diseases	57 (49.6)	58 (50.4)	115 (48.9)
Kidney and urinary tract	35 (47.9)	38 (52.1)	73 (31.1)
Heart disease	43 (62.3)	26 (37.7)	69 (29.4)
Diabetes mellitus	23 (39.7)	35 (60.3)	58 (24.7)
Gastrointestinal disease	30 (62.5)	18 (37.5)	48 (20.4)
Neurologic disease	23 (53.5)	20 (46.5)	43 (18.3)
Hypertension	20 (54.1)	17 (45.9)	37 (15.7)
Musculoskeletal disease	19 (51.4)	18 (48.6)	37 (15.7)

**Table 2:** Skin disorders distribution according to age group

Disease group	Age group (years old)		Total number of cases (%)	*P*-value

	60.69 (%)	≥ 70 (%)		
Infection	79 (39.5)	36 (29.8)	115 (35.8)	0.078^a^
Dermatitis	41 (20.5)	29 (24)	70 (21.8)	0.466^a^
Ulcer	21 (10.5)	20 (16.5)	41 (12.8)	0.117^b^
Age-related skin changes	17 (8.5)	10 (8.3)	27 (8.4)	0.941^b^
Pruritus	10 (5)	4 (3.3)	14 (4.4)	0.471^a^
Erythrosquamous dermatosis	1 (0.5)	1 (0.8)	2 (0.6)	0.613^b^
Drug eruptions	4 (2)	3 (2.5)	7 (2.2)	0.530^b^
Vascular disease	12 (6)	5 (4.1)	17 (5.3)	0.469^a^
Autoimmune vesico-bullous disease	4 (2)	7 (5.8)	11 (3.4)	0.070^b^
Benign neoplasms	0 (0)	1 (0.8)	1 (0.3)	0.377^a^
Metastatic tumour of the skin and malignant skin neoplasm	3 (1.5)	1 (0.8)	4 (1.2)	0.515^b^
Pyoderma	2 (1)	0 (0)	2 (0.6)	0.387^b^
Amyloidosis	1 (0.5)	0 (0)	1 (0.3)	0.623^b^
Disorders of pigmentation	5 (2.5)	3 (2.5)	8 (2.5)	0.648^a^
Disorders affecting the sweat duct	0 (0)	1 (0.8)	1 (0.3)	0.377^b^

Notes:

a chi-square test;

b Fisher’s exact test; statistically significant values *P* < 0.05

**Table 3:** Skin disorders distribution in male and female group

	Gender		Total number of cases	*P*-value

	Male (%)	Female (%)		
Infection	49 (29.7)	66 (42.3)	115 (35.8)	0.019^a^
Dermatitis	44 (26.7)	26 (16.7)	70 (21.8)	0.030^a^
Ulcer	18 (10.9)	23 (14.7)	41 (12.8)	0.304^a^
Age-related skin changes	15 (9.1)	12 (7.7)	27 (8.4)	0.652^b^
Pruritus	10 (6.1)	4 (2.6)	14 (4.4)	0.125^a^
Erythrosquamous dermatosis	1 (0.6)	1 (0.6)	2 (0.6)	0.737^b^
Drug eruptions	4 (2.4)	3 (1.9)	7 (2.2)	0.531^b^
Vascular disease	10 (6.1)	7 (4.5)	17 (5.3)	0.529^a^
Autoimmune vesico-bullous disease	4 (2.4)	7 (4.5)	11 (3.4)	0.310^a^
Benign neoplasms	1 (0.6)	0 (0)	1 (0.3)	0.514^b^
Metastatic tumour of the skin and malignant skin neoplasm	3 (1.8)	1 (0.6)	4 (1.2)	0.333^b^
Pyoderma	2 (1.2)	0 (0)	2 (0.6)	0.263^b^
Amyloidosis	1 (0.6)	0 (0)	1 (0.3)	0.514^b^
Disorders of pigmentation	3 (1.8)	5 (3.2)	8 (2.5)	0.331^b^
Disorders affecting the sweat duct	0 (0)	1 (0.6)	1 (0.3)	0.486^b^

Notes:

a chi-square test;

b Fisher’s exact test; statistically significant values *P* < 0.05
